# Trends by Acuity for Emergency Department Visits and Hospital Admissions in California, 2012 to 2022

**DOI:** 10.1001/jamanetworkopen.2023.48053

**Published:** 2023-12-18

**Authors:** Talia Ruxin, Madeline Feldmeier, Newton Addo, Renee Y. Hsia

**Affiliations:** 1 School of Medicine, University of California, San Francisco; 2 Department of Emergency Medicine, University of California, San Francisco; 3 Philip R. Lee Institute for Health Policy Studies, San Francisco, California

## Abstract

This cohort study investigates trends in emergency department (ED) visits and hospital admissions from EDs in California by visit acuity from 2012 to 2022.

## Introduction

Emergency departments (EDs) are an integral component of the US health care system, as they care for all patients and provide an entry point.^1^ However, ED visits have outpaced population growth while the number of EDs has decreased.^2^ Between 2011 and 2021, California ED visits increased by 7.4% while the number of EDs decreased by 3.8%.^2^ Furthermore, from 2006 to 2019, high complexity treat-and-release ED visits billed as high intensity increased from 4.8% to 19.2% nationally.^3^

Knowing the proportion of ED visits by acuity resulting in hospital admission may provide benchmarking trends for adequate hospital capacity and planning. Therefore, we investigated trends in California ED visits and hospital admissions from EDs by acuity from January 1, 2012, to December 31, 2022.

## Methods

This retrospective cohort study used publicly available ED visit data from the California Department of Healthcare Access and Information (HCAI), which mandates reporting from all hospitals in California.^4^ The University of California, San Francisco, Institutional Review Board approved the study and waived the need for informed consent owing to the use of publicly available data. The study followed the STROBE reporting guideline.

We defined ED visit acuity using *Current Procedural Technology* (*CPT*) codes based on classifications from the California HCAI. We stratified ED visits as nonurgent (99281), urgent (99282), moderate (99283), severe (99284), and critical (99285 and 99291) and as resulting in discharge or hospital admission. Data were analyzed using R, version 4.1 (R Project for Statistical Computing). Changes in the proportion of visits by *CPT* code were assessed with a χ^2^ trend test, and linear patterns in visits and admission were assessed using linear regression models. Two-sided *P* < .05 indicated statistical significance.

## Results

The overall number of ED visits grew from 12.5 to 14.3 million from 2012 to 2022 (14.0% [95% CI, 2.3%-28.7%]) (Table). During the study period, nonurgent visits decreased by −55.2% (95% CI, −61.3% to −47.0%) (Figure), whereas severe visits increased by 34.8% (95% CI, 20.9%-52.3%), and critical visits increased by 75.8% (95% CI, 62.5%-91.4%).

**Table.** ED Visits and Hospital Admissions From EDs by Visit Acuity, 2012 to 2022

ED visit acuity (*CPT* code)	Study year											Change 2012 to 2022, % (95% CI)	*P* value^a^
	2012	2013	2014	2015	2016	2017	2018	2019	2020	2021	2022		
**ED visits, No.**													
Nonurgent (99281)	796 173	800 986	801 400	808 106	776 707	733 601	703 543	450 142	316 722	336 071	356 464	−55.2 (−61.3 to 47.0)	<.001
Urgent (99282)	2 014 199	2 045 532	2 065 657	2 170 099	2 262 902	2 103 459	2 058 891	1 826 148	1 310 505	1 520 222	1 652 470	−18.0 (−27.9 to −4.8)	.01
Moderate (99283)	4 492 906	4 684 705	4 884 701	5 191 179	5 315 877	5 433 919	5 074 777	4 749 307	3 643 358	3 848 953	4 440 223	−1.2 (−16.6 to 14.3)	.17
Severe (99284)	3 063 915	3 101 587	3 349 631	3 550 280	3 595 306	3 808 953	3 793 604	4 399 317	3 434 760	3 799 651	4 130 943	34.8 (20.9 to 52.3)	.006
Critical (99285 and 99291)	2 040 242	1 986 544	2 260 799	2 404 990	2 537 614	2 756 110	3 057 700	3 374 000	3 084 971	3 375 539	3 586 523	75.8 (62.5 to 91.4)	<.001
Total^b^	12 505 718	12 722 085	13 436 083	14 198 173	14 560 356	14 928 933	14 781 546	14 876 653	11 860 597	12 944 692	14256171	14.0 (2.3 to 28.7)	.60
**Hospital admissions from EDs, No.**													
Nonurgent (99281)	8467	5271	6531	4361	6556	3527	3855	2838	5764	7330	1616	−80.9 (−85.1 to −73.4)	.12
Urgent (99282)	18 166	16 946	26 000	22 343	22 196	12 992	10 516	16 450	21 792	32 006	10 040	−44.7 (−62.9 to 8.5)	.83
Moderate (99283)	152 571	159 505	184 464	171 230	222 980	190 795	136 946	147 554	157 194	170 265	156 051	2.3 (−15.2 to 28.9)	.57
Severe (99284)	473 478	481 497	459 033	437 579	419 558	382 578	319 464	355 526	324 235	381 340	333 236	−29.6 (−34.8 to −23.5)	<.001
Critical (99285 and 99291)	1 075 460	1 054 890	1 095 615	1 149 611	1 173 720	1 249 622	1 332 966	1 332 997	1 259 379	1 326 255	1 345 735	25.1 (19.0 to 31.9)	<.001
Total	1 728 142	1 718 109	1 771 643	1 785 124	1 845 010	1 839 514	1 803 747	1 855 365	1 768 364	1 917 196	1 936 226	12.0 (8.6 to 15.7)	.002
**Hospital admissions from EDs, %**													
Nonurgent (99281)	1.1	0.7	0.8	0.5	0.8	0.5	0.5	0.6	1.8	2.2	0.5	−57.4 (−74.7 to 34.8)	.35
Urgent (99282)	0.9	0.8	1.3	1.0	1.0	0.6	0.5	0.9	1.7	2.1	0.6	−32.6 (−60.1 to 115)	.44
Moderate (99283)	3.4	3.4	3.8	3.3	4.2	3.5	2.7	3.1	4.3	4.4	3.5	3.5 (−13.9 to 29.7)	.46
Severe (99284)	15.5	15.5	13.7	12.3	11.7	10.0	8.4	8.1	9.4	10.0	8.1	−47.8 (−52.7 to −41.8)	<.001
Critical (99285 and 99291)	52.7	53.1	48.5	47.8	46.3	45.3	43.6	39.5	40.8	39.3	37.5	−28.8 (−30.7 to −26.8)	<.001

Abbreviations: *CPT*, *Current Procedural Terminology*; ED, emergency department.

^a^ *P* values are related to testing for linear trend.

^b^ Totals do not equal the sum by acuity due to visits missing CPT codes.

**Figure.**
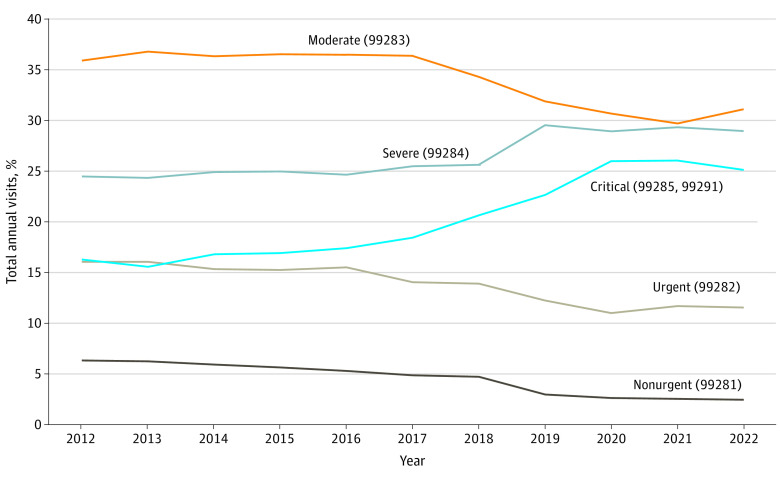
Emergency Department Visits by Acuity Acuity is expressed as *Current Procedural Terminology* codes (given parenthetically).

Likewise, the annual number of ED visits resulting in hospital admission increased by 12.0% (95% CI, 8.6%-15.7%). This increase was not equally distributed by acuity. The proportion of hospital admissions from nonurgent ED visits decreased from 1.1% in 2012 to 0.5% in 2022 (−54.5% [95% CI, −74.7% to −34.8%]). Notably, the proportion of hospital admissions from severe ED visits decreased from 15.5% in 2012 to 8.1% in 2022 (−47.8% [95% CI, −52.7% to −41.8%]), even as the proportion of these ED visits increased in that time. The proportion of critical ED visits resulting in admission decreased from 52.7% in 2012 to 37.5% in 2022 (−28.8% [95% CI, −30.7% to −26.8%]).

## Discussion

We found that from 2012 to 2022, the increase in severe and critical ED visits outpaced corresponding rates of hospital admissions from EDs. Severe visits rose by 34.8% and critical visits by 75.8%, aligning with findings of previous studies^5^ and suggesting that patients may be presenting with higher-acuity conditions. However, this observed rise in high-acuity ED visits was not met by a rise in hospital admissions; the proportion of severe and critical ED visits resulting in admission decreased from 15.5% to 8.1% and from 52.7% to 37.5%, respectively.

The findings of this cohort study raise questions about whether the increase in high-acuity ED visits can be fully explained by sicker patients or whether external factors (eg, more stringent hospital admissions criteria, changing demographics, and shifting practice patterns^3,6^) and/or upcoding (ie, documenting lower-acuity visits as higher-acuity, yielding higher payout) are substantial contributors. Study limitations include our use of administrative data, which do not have the granularity of clinical data or medical charts. These findings may inform policy makers and health care stakeholders when determining ED funding and resource allocation.

## Notes

Supplement.
Data Sharing Statement
