# Supplementary material for: Trends by Acuity for Emergency Department Visits and Hospital Admissions in California, 2012 to 2022
Source: JAMA Netw Open. 2023 Dec 18;6(12):e2348053. doi: 10.1001/jamanetworkopen.2023.48053 (PMC10728761; doi:10.1001/jamanetworkopen.2023.48053)
Supplement: Supplement. — Data Sharing Statement [file jamanetwopen-e2348053-s001.pdf]

Data Sharing Statement

Ruxin. Trends by Acuity for Emergency Department Visits and Hospital Admissions in California, 2012 to 2022. *JAMA Netw Open*. Published December 18, 2023. doi:10.1001/jamanetworkopen.2023.48053

Data
Data available: No
